# Effect of general anesthesia vs. local anesthesia and collateral status on outcomes in anterior circulation occlusion

**DOI:** 10.3389/fneur.2025.1665185

**Published:** 2025-11-25

**Authors:** Guojie Chen, Rundong Chen, Tianxiang Gao, Lijun Wang, Hongye Xu, Wen Yin, Yu Gao, Lei Zhang, Yongxin Zhang, Pengfei Xing, Pengfei Yang, Zifu Li, Yongwei Zhang, Jianmin Liu

**Affiliations:** 1 Neurovascular Center, Changhai Hospital, Naval Medical University, Shanghai, China; 2 Department of Anesthesiology, Changhai Hospital, Naval Medical University, Shanghai, China

**Keywords:** acute ischemic stroke, endovascular treatment, anesthesia, collateral status, propensity score matching, functional independence

## Abstract

**Background and objectives:**

The impact of anesthesia type on outcomes following endovascular thrombectomy (EVT) remains controversial. Collateral status assessed through perfusion imaging may provide critical insights for optimizing anesthesia strategies during EVT.

**Methods:**

In this retrospective cohort study, functional outcomes after EVT (measured by the modified Rankin Scale score) were compared between general anesthesia (GA) vs. local anesthesia (LA) using a propensity score-matched model. The association between the hypoperfusion intensity ratio (HIR, defined as Tmax > 10s/Tmax > 6 s) and outcomes was evaluated through weighted multivariate logistic regression, with potential non-linearity explored using restricted cubic spline (RCS) regression. To validate the findings, five analytical approaches were applied, including propensity score matching, multivariate logistic modeling adjusted for all covariates, inverse probability of treatment weighting (IPTW), and doubly robust estimations with and without adjustments for unbalanced covariates.

**Results:**

A total of 702 patients were included, with 327 (46.6%) receiving GA and 375 (53.4%) receiving LA. Propensity score matching achieved balanced baseline characteristics (*p* > 0.05). Among patients with good collateral status (HIR <0.4), GA was associated with worse functional outcomes (mRS 0–2: 49% vs. 70%; OR 2.88, 95% CI: 1.29–6.43). In patients with poor collateral status, outcomes were comparable between GA and LA (mRS 0–2: 50% vs. 59%; OR 1.73, 95% CI: 0.92–3.27). All five statistical models yielded consistent results.

**Conclusions:**

There is an association between general anesthesia and poorer functional prognosis in patients with well-developed collateral circulation after endovascular thrombectomy (EVT). HIR may serve as a useful marker for anesthesia selection and triage in EVT.

**Classification of evidence:**

This study provides Class III evidence that use of GA is associated with worse functional outcome in patients with good collateral that undergoing EVT.

## Introduction

Endovascular thrombectomy (EVT) has become the first-line treatment for patients with acute ischemic stroke caused by large vessel occlusion (AIS-LVO) (1), offering significant improvements in clinical outcomes. While EVT's efficacy is well-established (2), optimizing procedural variables, including anesthesia type, remains a critical area of investigation. Current evidence on the impact of anesthesia type—general anesthesia (GA) (3) vs. local anesthesia (LA) or conscious sedation (CS)—on functional outcomes is conflicting. Individual-level meta-analysis of the HERMES collaboration demonstrated worse outcomes with GA (3), whereas data from randomized trials, including the GOLIATH, SIESTA, and ANSTROKE studies, suggested improved or comparable outcomes with GA (4, 5). Most recently, the AMETIS trial reported similar results, further complicating the narrative.

Robust pial collaterals have been identified as a critical imaging biomarker for improved EVT outcomes (6), contributing to slower infarct progression and enhanced hemodynamic stability in ischemic areas. Patients with good collateral status may benefit from preserved cerebral blood flow, even in the setting of ischemia. Anesthesia choice can modulate these dynamics: LA/CS, being less invasive, is less likely to cause procedural hypotension, which could impair collateral circulation (7, 8). Conversely, GA provides airway protection and facilitates procedural control but may introduce hemodynamic instability. Thus, understanding how collateral status interacts with anesthesia type to influence outcomes is crucial for tailoring treatment strategies.

We hypothesize that the effect of anesthesia type on EVT outcomes is modulated by collateral status, as reflected by the hypoperfusion intensity ratio (HIR). This ratio, derived from perfusion imaging, reflects the extent of ischemic compromise and could serve as a surrogate marker to guide anesthesia selection. In this study, we aimed to investigate the relationship between HIR and functional outcomes after EVT and determine whether HIR could help identify patients who would benefit from a specific anesthesia approach.

## Methods

### Study population

This single-center, retrospective observational cohort study included 948 consecutive patients diagnosed with acute ischemic stroke (AIS) due to large-vessel occlusion (LVO) in the anterior circulation who underwent endovascular thrombectomy (EVT) between January 2018 and December 2022. Patients were eligible if they were aged ≥18 years, had anterior circulation occlusion [intracranial internal carotid artery (ICA) or first/second segment of the middle cerebral artery (MCA)] confirmed by computed tomography angiography (CTA), and had available hypoperfusion intensity ratio (HIR) evaluation. Informed consent was not required, as only anonymized data collected prospectively during routine clinical care were analyzed, per local legislation.

### Imaging evaluation

All patients underwent non-contrast CT, CT angiography, and CT perfusion imaging before EVT, processed using iSchemaView RAPID software. Images were not reprocessed for this study. HIR was calculated as the ratio of brain volume with time-to-max (Tmax) delay >10 s to the volume with Tmax >6 s (9, 10). Good collateral status was defined as HIR between 0 and 0.4, while poor collateral status was defined as HIR between 0.4 and 1.0.

### Anesthesia regimens

The recanalization time after thrombectomy is of great significance for the prognosis of stroke patients. For most patients, surgeons first perform thrombectomy under local anesthesia. During cerebral angiography, the need for general anesthesia is determined based on the location and size of the thrombus. Among the patients who received general anesthesia in our study, some had undergone local anesthesia prior to general anesthesia. For patients undergoing general anesthesia, we administer propofol, sufentanil, and cisatracurium for anesthetic induction in accordance with drug package inserts and clinical guidelines. For anesthetic maintenance, propofol, remifentanil, and sevoflurane are used. All these drugs can affect the hemodynamic stability of patients. When a patient's mean arterial pressure drops by more than 30%, anesthesiologists will administer vasoactive drugs to maintain the patient's blood pressure stability.

### Patient characteristics

Baseline characteristics included age, sex, body mass index (BMI), history of hypertension, diabetes, hyperlipidemia, atrial fibrillation, prior stroke, smoking, alcohol consumption, systolic blood pressure (SBP), and diastolic blood pressure (DBP) at admission. Clinical assessments included admission National Institutes of Health Stroke Scale (NIHSS) score, Alberta Stroke Program Early CT Score (ASPECTS), stroke etiology, time from symptom onset to reperfusion, anesthesia method, and recanalization status. The extended Thrombolysis in Cerebral Infarction (eTICI) score was used to assess reperfusion, ranging from 0 (no reperfusion) to 3 (complete reperfusion) (11).

### Outcomes

The primary outcome was favorable functional outcome, defined as a modified Rankin Scale (mRS) score of 0–2 at 90 days. Outcomes were assessed by experienced investigators blinded to baseline information via face-to-face or telephone interviews using a standardized protocol. Secondary outcomes included mRS scores of 0–1 and 0–3 at 90 days, symptomatic intracranial hemorrhage (sICH) within 72 h, NIHSS score at 7 days, stroke-associated pneumonia, early neurological deterioration, and 90-day mortality.

### Statistical analysis

Patients were stratified into general anesthesia (GA) and local anesthesia (LA) groups. Continuous variables were summarized as mean ± standard deviation (SD) or median with interquartile range (IQR), while categorical variables were presented as counts and percentages. Student's *t*-test was used for normally distributed continuous variables, Mann–Whitney *U*-test for non-normally distributed variables, and Chi-squared test for categorical variables. Baseline variables associated with outcomes (*p* < 0.05) in univariate analysis, including sex, TOAST classification, atrial fibrillation, baseline NIHSS, perfusion mismatch, tirofiban use, and time from symptom onset to reperfusion, were included in multivariate logistic regression models (12, 13).

Restricted cubic spline (RCS) regression was used to assess the nonlinear relationship between HIR and outcomes, with knots set at the 10th, 50th, and 90th percentiles of the HIR distribution. Adjustments were made for clinically relevant covariates based on prior knowledge. Additionally, doubly robust estimation methods were applied, combining multivariate regression models with propensity score models to evaluate the causal effect of anesthesia type on outcomes. This approach ensures unbiased effect estimates if at least one model is correctly specified.

Five inferential models were utilized: (1) a multivariate logistic regression model adjusted for all covariates; (2) a propensity score matching model; (3) an inverse probability of treatment weighting (IPTW) model; (4) a doubly robust model adjusted for all covariates; and (5) a doubly robust model adjusted for unbalanced covariates (14). Statistical analyses were conducted using R software (version 4.3.2; R Foundation for Statistical Computing, Vienna, Austria). A two-sided *p*-value <0.05 was considered statistically significant.

## Results

**Figure 1:**
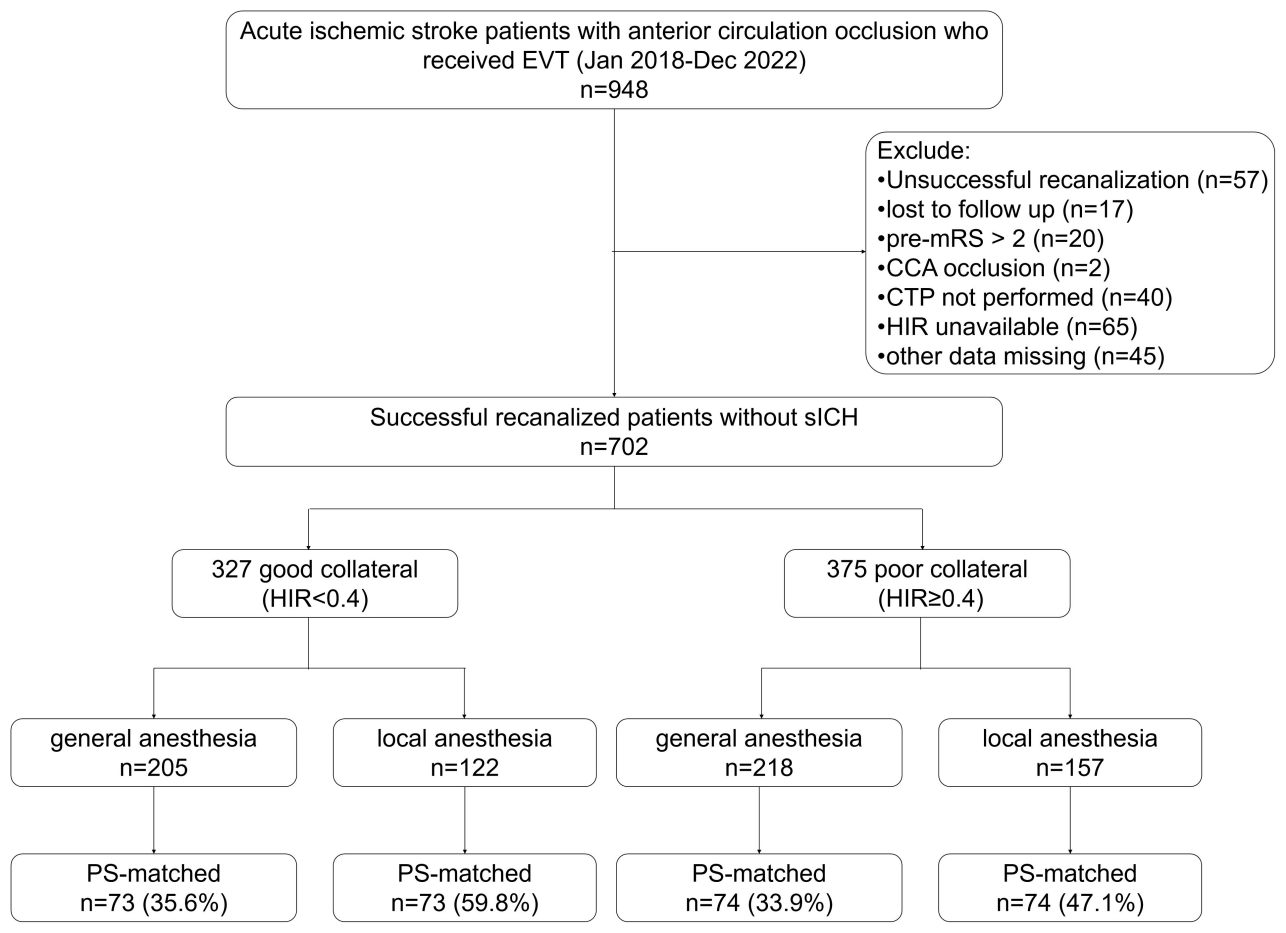
Flowchart of patient selection and group allocation.

A total of 948 patients with anterior circulation occlusion underwent endovascular thrombectomy (EVT) during the study period, of whom 702 met the inclusion criteria. Detailed reasons for exclusion are presented in Figure 1. Among the included patients, 327 exhibited good collateral circulation (HIR <0.4), while 375 had poor collateral circulation (HIR ≥ 0.4). In the good collateral cohort, 208 patients received general anesthesia (GA), and 122 underwent local anesthesia (LA) or conscious sedation (CS). In the poor collateral cohort, 218 and 157 patients were treated under GA and LA/CS, respectively.

Before propensity score matching, significant differences in baseline characteristics, including sex, stroke etiology, history of hypertension and atrial fibrillation, mismatch volume, tirofiban use, and time from symptom onset to reperfusion, were observed in the good collateral cohort (*p* < 0.05, Table 1). After propensity score matching, these variables were balanced in both the good and poor collateral cohorts (Supplementary Table 1).

**Table 1:** Baseline characteristics before and after propensity score matching in good collateral cohorts (HIR <0.4).

**Baseline**	**Before matching**				**After matching**			
	**Overall (*****N*** = **327)**	**General_anesthesia (*****N*** = **205)**	**Local_anesthesia (*****N*** = **122)**	**SMD**	**Overall (*****N*** = **146)**	**General_anesthesia (*****N*** = **73)**	**Local_anesthesia (*****N*** = **73)**	**SMD**
**Demographic data**								
**Age, median (IQR)**	67.00 [59.00, 74.50]	66.00 [59.00, 74.00]	67.00 [60.00, 75.00]	0.085	67.00 [58.00, 75.00]	67.00 [58.00, 76.00]	67.00 [58.00, 75.00]	0.034
Female, (%)	112 (34.25)	60 (29.27)	52 (42.62)	0.281	56 (38.36)	30 (41.10)	26 (35.62)	0.113
BMI, median (IQR)	23.66 [21.92, 25.71]	24.22 [22.04, 25.71]	23.29 [21.15, 25.80]	0.137	23.88 (3.01)	24.24 (2.88)	23.53 (3.12)	0.238
Systolic blood pressure, median (IQR)	132.00 [120.00, 148.00]	134.00 [120.00, 151.00]	130.50 [116.00, 142.75]	0.197	132.50 [121.00, 148.00]	129.00 [117.00, 148.00]	135.00 [124.00, 148.00]	0.139
**Diastolic blood pressure, median (IQR)**	78.00 [71.00, 87.00]	78.00 [71.00, 87.00]	78.00 [70.25, 85.00]	0.106	79.50 [72.00, 89.00]	80.00 [71.00, 90.00]	79.00 [74.00, 89.00]	0.029
**TOAST, (%)**								
Atherosclerosis	151 (46.18)	105 (51.22)	46 (37.70)	0.102	68 (46.58)	33 (45.21)	35 (47.95)	0.148
Cardioembolism	101 (30.89)	52 (25.37)	49 (40.16)		45 (30.82)	21 (28.77)	24 (32.88)	
Other	14 (4.28)	9 (4.39)	5 (4.10)		6 (4.11)	3 (4.11)	3 (4.11)	
Undetermined	61 (18.65)	39 (19.02)	22 (18.03)		27 (18.49)	16 (21.92)	11 (15.07)	
**Medical history**								
Wake up stroke, (%)	73 (22.32)	42 (20.49)	31 (25.41)	0.117	32 (21.92)	13 (17.81)	19 (26.03)	0.2
History of hypertension, (%)	210 (64.22)	143 (69.76)	67 (54.92)	0.31	96 (65.75)	46 (63.01)	50 (68.49)	0.116
History of diabetes mellitus, (%)	87 (26.61)	57 (27.80)	30 (24.59)	0.073	44 (30.14)	23 (31.51)	21 (28.77)	0.06
History of smoke, (%)	139 (42.51)	92 (44.88)	47 (38.52)	0.129	63 (43.15)	28 (38.36)	35 (47.95)	0.195
History of alcohol consumption, (%)	66 (20.18)	40 (19.51)	26 (21.31)	0.045	32 (21.92)	14 (19.18)	18 (24.66)	0.133
Previous ischemic stroke, (%)	86 (26.30)	54 (26.34)	32 (26.23)	0.003	46 (31.51)	22 (30.14)	24 (32.88)	0.059
History of atrial fibrillation, (%)	104 (31.80)	54 (26.34)	50 (40.98)	0.314	49 (33.56)	23 (31.51)	26 (35.62)	0.087
**History of hyperlipemia, (%)**	69 (21.10)	42 (20.49)	27 (22.13)	0.04	31 (21.23)	15 (20.55)	16 (21.92)	0.034
**Baseline assessments**								
History of coronary heart disease, (%)	47 (14.37)	26 (12.68)	21 (17.21)	0.127	24 (16.44)	15 (20.55)	9 (12.33)	0.223
Previous anticoagulants medication, (%)	20 (6.12)	9 (4.39)	11 (9.02)	0.186	10 (6.85)	4 (5.48)	6 (8.22)	0.109
Previous antiplatelet medication, (%)	57 (17.43)	31 (15.12)	26 (21.31)	0.161	28 (19.18)	13 (17.81)	15 (20.55)	0.07
**Pre-morbidity mRS, (%)**								
0	296 (90.52)	188 (91.71)	108 (88.52)	0.067	131 (89.73)	66 (90.41)	65 (89.04)	0.063
1	22 (6.73)	11 (5.37)	11 (9.02)		10 (6.85)	5 (6.85)	5 (6.85)	
2	9 (2.75)	6 (2.93)	3 (2.46)		5 (3.42)	2 (2.74)	3 (4.11)	
Baseline NIHSS score, median (IQR)	13.00 [8.00, 18.00]	13.00 [8.00, 18.00]	11.00 [7.00, 17.00]	0.166	12.50 [7.00, 18.00]	14.00 [9.00, 18.00]	10.00 [6.00, 16.00]	0.373
ASPECTS, median (IQR)	9.00 [7.00, 10.00]	9.00 [7.00, 10.00]	9.00 [7.00, 10.00]	0.041	9.00 [7.00, 10.00]	9.00 [7.00, 9.00]	9.00 [7.00, 10.00]	0.099
**Occlusion site, (%)**								
ICA	117 (35.78)	75 (36.59)	42 (34.43)	0.128	52 (35.62)	27 (36.99)	25 (34.25)	0.155
ACA	7 (2.14)	6 (2.93)	1 (0.82)		4 (2.74)	3 (4.11)	1 (1.37)	
M1	176 (53.82)	112 (54.63)	64 (52.46)		76 (52.05)	39 (53.42)	37 (50.68)	
M2	26 (7.95)	12 (5.85)	14 (11.48)		14 (9.59)	4 (5.48)	10 (13.70)	
M3	1 (0.31)	0 (0.00)	1 (0.82)		0 (0.00)	0 (0.00)	0 (0.00)	
Ischemic core volume (ml), median (IQR)	0.00 [0.00, 11.00]	0.00 [0.00, 8.00]	4.00 [0.00, 13.75]	0.157	0.00 [0.00, 8.00]	0.00 [0.00, 6.00]	0.00 [0.00, 13.00]	0.273
Mismatch volume (ml), median (IQR)	113.00 [72.50, 168.50]	119.00 [79.00, 178.00]	101.50 [62.50, 150.00]	0.2	101.50 [66.25, 154.00]	109.00 [78.00, 159.00]	94.00 [52.00, 145.00]	0.202
Direct EVT	257 (78.59)	160 (78.05)	97 (79.51)	0.036	114 (78.08)	54 (73.97)	60 (82.19)	0.2
**BGC use, (%)**	77 (23.55)	42 (20.49)	35 (28.69)	0.191	39 (26.71)	19 (26.03)	20 (27.40)	0.031
**Per procedural GPIIb/IIIa receptor antagonist, (%)**	174 (53.21)	125 (60.98)	49 (40.16)	0.426	74 (50.68)	37 (50.68)	37 (50.68)	<0.001
**eTICI 2c/3 on final DSA**	250 (76.45)	161 (78.54)	89 (72.95)	0.131	109 (74.66)	55 (75.34)	54 (73.97)	0.031
Time from stroke onset to reperfusion	504.00 [317.50, 838.00]	549.00 [350.00, 910.00]	437.50 [267.50, 763.00]	0.096	454.00 [291.50, 834.75]	455.00 [293.00, 793.00]	441.00 [269.00, 840.00]	0.052
**Outcome**								
mRS 0–2 at 90d, (%)	196 (59.94)	111 (54.15)	85 (69.67)	0.324	87 (59.59)	36 (49.32)	51 (69.86)	0.428
mRS 0–1 at 90d, (%)	160 (48.93)	88 (42.93)	72 (59.02)	0.326	72 (49.32)	28 (38.36)	44 (60.27)	0.449
mRS 0–3 at 90d, (%)	230 (70.34)	134 (65.37)	96 (78.69)	0.3	105 (71.92)	45 (61.64)	60 (82.19)	0.47
Symptomatic intracranial hemorrhage, (%)	21 (6.42)	14 (6.83)	7 (5.74)	0.045	6 (4.11)	4 (5.48)	2 (2.74)	0.138
NIHSS score at 7 days	4.00 [2.00, 11.00]	4.00 [2.00, 13.00]	2.50 [1.00, 6.00]	0.356	3.00 [2.00, 9.00]	6.00 [2.00, 11.00]	2.00 [0.00, 6.00]	0.408
Stroke associated pneumonia, (%)	74 (22.63)	54 (26.34)	20 (16.39)	0.245	25 (17.12)	15 (20.55)	10 (13.70)	0.183
Early neurological deterioration, (%)	39 (11.93)	30 (14.63)	9 (7.38)	0.233	15 (10.27)	11 (15.07)	4 (5.48)	0.32
Mortality at 90d, (%)	40 (12.23)	32 (15.61)	8 (6.56)	0.291	14 (9.59)	10 (13.70)	4 (5.48)	0.282

Values are presented as mean (standard deviation) or median [Q1, Q3] for continuous variables and number (percentage) for categorical variables. Variables in bold have *p*-value <0.05.

**Figure 2:**
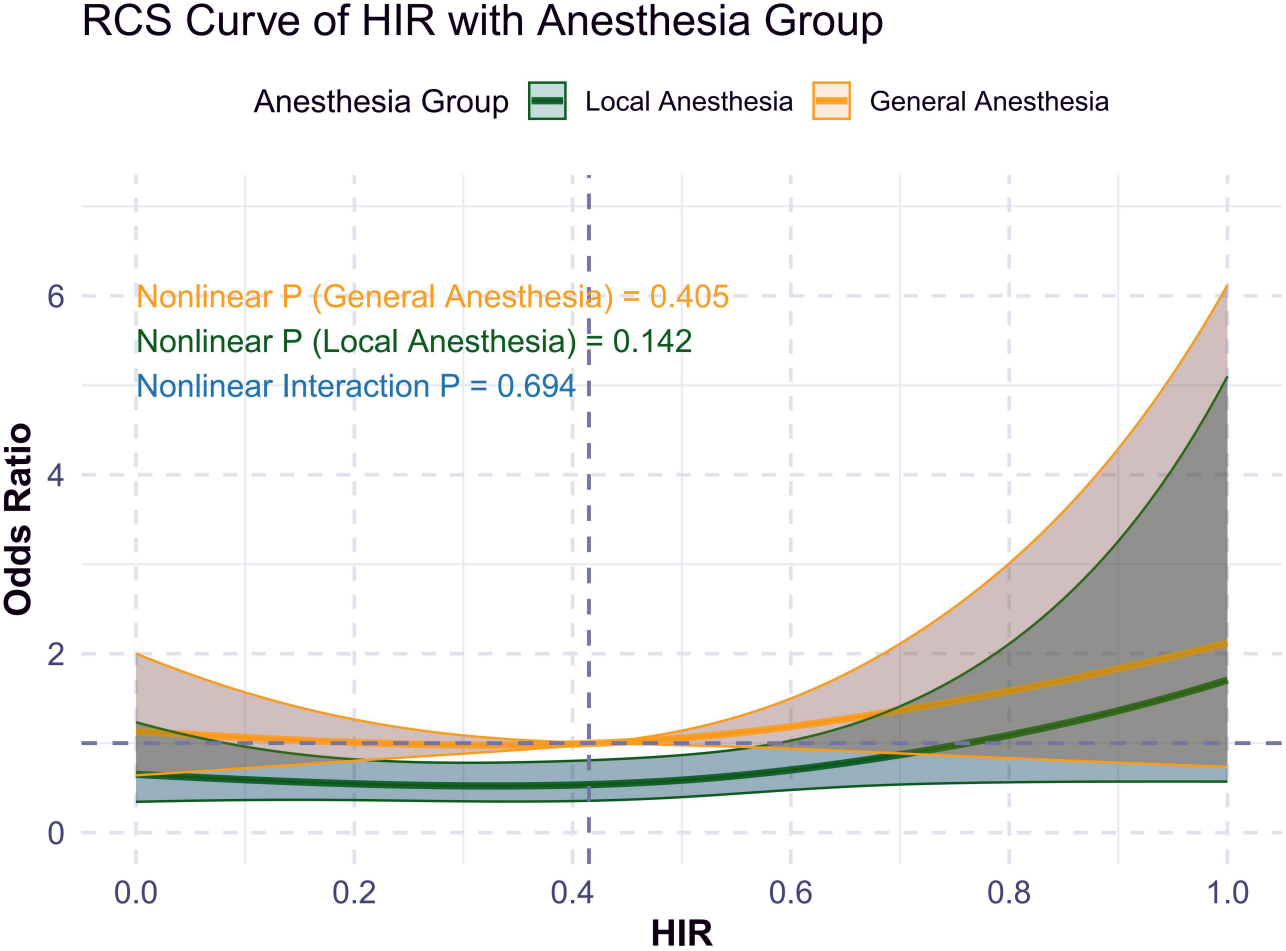
RCS curve of HIR and primary outcome (mRS 0-2) by anesthesia choice.

Restricted cubic spline (RCS) analysis demonstrated that higher HIR was associated with a lower probability of functional independence (mRS 0–2) at 90 days (unadjusted *p* < 0.01; adjusted *p* = 0.073; Supplementary Figure 1). This trend was consistent across GA and LA cohorts but did not reach statistical significance (Figure 2). There was no significant interaction between anesthesia type and functional independence (*p* = 0.649).

In the good collateral subgroup (HIR <0.4), GA was associated with worse functional outcomes compared to LA/CS. Specifically, the probability of achieving mRS 0–2 was significantly lower (49% vs. 70%; OR 2.88, 95% CI: 1.29–6.43). GA was also associated with worse outcomes in terms of mRS 0–1 (38% vs. 60%), mRS 0–3 (62% vs. 82%), and higher NIHSS scores at 7 days (median: 6 vs. 2; Table 2). In contrast, among patients with poor collateral circulation (HIR ≥ 0.4), outcomes were similar between GA and LA/CS, with comparable rates of mRS 0–2 (50% vs. 59%; OR 1.73, 95% CI: 0.92–3.27; Table 3).

**Table 2:** Primary outcome (mRS 0-2) with different models in patients with good collateral.

**Baseline**	***p*-value**	**Result**
**Propensity score matching [OR (95 CI)]** ^**1**^	<0.05	2.88 (1.29, 6.43)
**Multivariate logistic model adjusted with all covariates [OR (95 CI)]** ^**1**^	<0.01	2.27 (1.37, 3.84)
**Propensity score IPTW [OR (95 CI)]** ^**1**^	<0.001	2.37 (1.69, 3.34)
**Doubly robust estimation with all covariates [OR (95 CI)]** ^**1**^	<0.01	2.37 (1.42, 3.98)
**Doubly robust estimation with unbalanced covariates [OR (95 CI)]** ^**1**^	<0.01	2.34 (1.40, 3.91)

Statistical analyses of different models with p-value <0.05 were displayed in bold. ^1^OR, odds ratio; CI, confidence interval.

**Table 3:** Primary outcome (mRS 0–2) with different models in patients with poor collateral.

**Baseline**	***p*-value**	**Result**
Propensity score matching [OR (95 CI)]^1^	0.155	1.67 (0.88, 3.16)
**Multivariate logistic model adjusted with all covariates [OR (95 CI)]** ^**1**^	<0.05	1.66 (1.02, 2.73)
**Propensity score IPTW [OR (95 CI)]** ^**1**^	<0.01	1.72 (1.23, 2.40)
**Doubly robust estimation with all covariates [OR (95 CI)]** ^**1**^	<0.05	1.72 (1.04, 2.83)
**Doubly robust estimation with unbalanced covariates [OR (95 CI)]** ^**1**^	<0.05	1.72 (1.04, 2.84)

Statistical analyses of different models with *p*-value <0.05 were displayed in bold. ^1^OR, odds ratio; CI, confidence interval.

Sensitivity analyses using five inferential models—multivariate logistic regression adjusted for all covariates, propensity score-based IPTW, and doubly robust estimations with and without adjustment for unbalanced covariates—produced consistent results. Across all models, GA was associated with worse functional outcomes in the good collateral subgroup, while no significant differences were observed in the poor collateral subgroup.

## Discussion

This study demonstrates that the hypoperfusion intensity ratio (HIR) is strongly associated with functional outcomes following endovascular thrombectomy (EVT) in anterior circulation occlusions. Notably, we observed a differential effect of anesthesia type based on collateral status: general anesthesia (GA) was associated with worse outcomes in patients with good collateral circulation (HIR <0.4), whereas no significant differences were found between GA and local anesthesia (LA) in patients with poor collateral circulation (HIR ≥ 0.4). These findings highlight the potential of HIR as a surrogate marker to guide anesthesia selection in EVT.

HIR, a perfusion imaging-derived biomarker, has proven to be a reliable indicator of microvascular collateral flow and tissue viability in ischemic stroke (15). Robust collateral circulation, reflected by low HIR values, is critical for preserving microvascular integrity, reducing infarct growth, and enhancing tissue salvage (16). This study underscores the value of HIR not only as a prognostic marker but also as a tool for tailoring procedural strategies, such as anesthesia choice (17).

Most drugs involved in general anesthesia can affect hemodynamics to varying degrees. In patients with well-developed collateral circulation, vasodilation in non-ischemic regions may shunt blood away from the ischemic penumbra after general anesthesia (18). Additionally, certain anesthetic drugs and perioperative factors (such as hypotension, hypercapnia, and inflammatory cascade reactions) may alter the permeability of the blood-brain barrier. Even in the presence of good collateral circulation, this can increase susceptibility to reperfusion injury (19).

The finding that GA is associated with worse outcomes in patients with good collateral circulation aligns with the hypothesis that GA-induced hemodynamic instability, such as hypotension, may disproportionately affect ischemic regions with higher perfusion reserves. In contrast, for patients with poor collateral circulation, the limited perfusion reserve may render the protective effects of LA less pronounced, resulting in similar outcomes across both anesthesia types (8, 20–22).

Our results are consistent with prior studies demonstrating worse functional outcomes with GA, including a pooled analysis of randomized trials and prospective cohorts where GA was independently associated with lower odds of good outcomes (adjusted OR: 0.64, *p* = 0.021) and higher rates of neurological deterioration (adjusted OR: 2.10, *p* = 0.045). However, these studies did not fully account for the role of collateral status. Interestingly, while prior analyses suggested no difference in outcomes based on collateral status, our study found a robust and statistically significant association between GA and worse outcomes in patients with good collateral, supported across all statistical models.

This distinction may reflect differences in study design, imaging methodologies, or statistical approaches. Importantly, no prior randomized trials have specifically evaluated the interaction between imaging biomarkers such as HIR and anesthesia type, further emphasizing the novelty and clinical relevance of our findings.

From a clinical perspective, our findings suggest that anesthesia strategies for EVT should be tailored based on pre-procedural imaging markers, such as HIR. Patients with good collateral status may benefit from avoiding GA when feasible, as LA appears less likely to disrupt hemodynamics. Integrating HIR into routine EVT workflows could enhance patient selection and optimize outcomes.

This study has several limitations. As a single-center, retrospective analysis, it is subject to potential selection bias and residual confounding, even with the use of propensity score matching and doubly robust statistical methods. The non-randomized design limits causal inference, and unmeasured confounders, such as the indication for GA in patients with more severe conditions, cannot be excluded. Intraoperative hemodynamic parameters were not recorded in this study. Blood pressure decrease caused by anesthetic drugs, the duration of intraoperative hypotension, and fluctuations in MAP can all lead to poor neurological prognosis. Therefore, we cannot determine whether the observed association is caused by the anesthetic method or hemodynamic fluctuations. Moreover, in our study, vasoactive drugs were administered only when the MAP dropped by 30%, which may result in insufficient cerebral blood perfusion. This approach is likely to lead to a poorer prognosis in patients undergoing GA. Furthermore, the skewed distribution of HIR may introduce bias in regression analyses, and HIR alone may not fully capture the complexity of collateral dynamics. Future research should focus on validating these findings in multicenter, prospective studies and randomized controlled trials. Specifically, studies should explore the interaction between imaging biomarkers, anesthesia type, and patient outcomes to establish evidence-based criteria for anesthesia selection. Mechanistic studies investigating how GA affects cerebral hemodynamics in different collateral states may further refine procedural protocols and improve EVT outcomes.

## Conclusions

This study identifies HIR as a key biomarker for predicting functional outcomes and guiding anesthesia strategies in EVT. Our findings highlight the need for personalized approaches to anesthesia selection, particularly for patients with good collateral circulation. Further validation in larger, randomized studies is warranted to confirm the role of HIR in optimizing EVT outcomes.

## Data Availability

The original contributions presented in the study are included in the article/Supplementary material, further inquiries can be directed to the corresponding authors.

## Supplementary material

Supplementary Figure 1 RCS curve of HIR and primary outcome (mRS 0–2).

Supplementary Table 1 Baseline characteristics before and after propensity score matching in poor collateral cohorts (HIR ≥ 0.4).

Supplementary Table 2 Secondary outcome (mRS 0–1) with different models for cohort.

Supplementary Table 3 Secondary outcome (mRS 0–3) with different models for cohort.

Supplementary Table 4 Analysis results of the secondary outcomes of the cohort.

## References

[1] Goyal M Menon BK van Zwam WH Dippel DW Mitchell PJ Demchuk AM . Endovascular thrombectomy after large-vessel ischaemic stroke: A meta-analysis of individual patient data from five randomised trials. Lancet. (2016) 387:1723–31. doi: 10.1016/S0140-6736(16)00163-X 26898852

[2] Campbell D Butler E Campbell RB Ho J Barber PA. General anesthesia compared with non-ga in endovascular thrombectomy for ischemic stroke: a systematic review and meta-analysis of randomized controlled trials. Neurology. (2023) 100:e1655–63. doi: 10.1212/WNL.0000000000207066 36797071 PMC10115505

[3] Campbell BCV van Zwam WH Goyal M Menon BK Dippel DWJ Demchuk AM . Effect of general anaesthesia on functional outcome in patients with anterior circulation ischaemic stroke having endovascular thrombectomy versus standard care: a meta-analysis of individual patient data. Lancet Neurol. (2018) 17:47–53. doi: 10.1016/S1474-4422(17)30407-6 29263006

[4] Schönenberger S Hendén PL Simonsen CZ Uhlmann L Klose C Pfaff JAR . Association of general anesthesia vs procedural sedation with functional outcome among patients with acute ischemic stroke undergoing thrombectomy: a systematic review and meta-analysis. JAMA. (2019) 322:1283–93. doi: 10.1001/jama.2019.11455 31573636 PMC6777267

[5] Chabanne R Geeraerts T Begard M Balança B Rapido F Degos V . Outcomes after endovascular therapy with procedural sedation vs general anesthesia in patients with acute ischemic stroke: the ametis randomized clinical trial. JAMA Neurol. (2023) 80:474–83. doi: 10.1001/jamaneurol.2023.0413 37010829 PMC10071397

[6] Guenego A Marcellus DG Martin BW Christensen S Albers GW Lansberg MG . Hypoperfusion intensity ratio is correlated with patient eligibility for thrombectomy. Stroke. (2019) 50:917–22. doi: 10.1161/STROKEAHA.118.024134 30841821

[7] Anadani M Gory B Olivot JM Bourcier R Consoli A Boulouis G . The impact of general anesthesia versus non-general anesthesia on thrombectomy outcomes by occlusion location: Insights from the ETIS registry. J Neurosur. 2024:1–9. doi: 10.3171/2024.5.JNS24199 39178480

[8] Feil K Herzberg M Dorn F Tiedt S Küpper C Thunstedt DC . General anesthesia versus conscious sedation in mechanical thrombectomy. J Stroke. (2021) 23:103–12. doi: 10.5853/jos.2020.02404 33600707 PMC7900389

[9] Sarraj A Albers GW Mitchell PJ Hassan AE Abraham MG Blackburn S . Thrombectomy outcomes with general vs nongeneral anesthesia: A pooled patient-level analysis from the extend-ia trials and select study. Neurology. (2023) 100:e336–47. doi: 10.1212/WNL.0000000000201384 36289001 PMC9869759

[10] Faizy TD Kabiri R Christensen S Mlynash M Kuraitis G Broocks G . Perfusion imaging-based tissue-level collaterals predict ischemic lesion net water uptake in patients with acute ischemic stroke and large vessel occlusion. J Cereb Blood Flow Metab. (2021) 41:2067–75. doi: 10.1177/0271678X21992200 33557694 PMC8327120

[11] Lapergue B Blanc R Costalat V Desal H Saleme S Spelle L . Effect of thrombectomy with combined contact aspiration and stent retriever vs stent retriever alone on revascularization in patients with acute ischemic stroke and large vessel occlusion: the aster2 randomized clinical trial. JAMA. (2021) 326:1158–69. doi: 10.1001/jama.2021.13827 34581737 PMC8479584

[12] Li Y Wang Y Jing Y Zhu Y Huang X Wang J . Visualization analysis of breast cancer-related ubiquitination modifications over the past two decades. Discov Oncol. (2025) 16:431. doi: 10.1007/s12672-025-02032-1 40163091 PMC11958930

[13] Wang Y Li Y Jing Y Yang Y Wang H Ismtula D . Tubulin alpha-1b chain was identified as a prognosis and immune biomarker in pan-cancer combing with experimental validation in breast cancer. Sci Rep. (2024) 14:8201. doi: 10.1038/s41598-024-58982-z 38589634 PMC11001892

[14] Cole SR Hernán MA. Constructing inverse probability weights for marginal structural models. Am J Epidemiol. (2008) 168:656–4. doi: 10.1093/aje/kwn164 18682488 PMC2732954

[15] Gensicke H Al-Ajlan F Fladt J Campbell BCV Majoie C Bracard S . Comparison of three scores of collateral status for their association with clinical outcome: the hermes collaboration. Stroke. (2022) 53:3548–56. doi: 10.1161/STROKEAHA.122.039717 36252099

[16] Lee JS Bang OY. Collateral status and outcomes after thrombectomy. Transl Stroke Res. (2023) 14:22–37. doi: 10.1007/s12975-022-01046-z 35687300

[17] Liebeskind DS Saber H Xiang B Jadhav AP Jovin TG Haussen DC . Collateral circulation in thrombectomy for stroke after 6 to 24 hours in the dawn trial. Stroke. (2022) 53:742–48. doi: 10.1161/STROKEAHA.121.034471 34727737

[18] Bellomo J Sebök M Stumpo V van Niftrik CHB Meisterhans D Piccirelli M . Blood oxygenation level–dependent cerebrovascular reactivity–derived steal phenomenon may indicate tissue reperfusion failure after successful endovascular thrombectomy. Transl Stroke Res. (2023) 16:207–16. doi: 10.1007/s12975-023-01203-y 37880561 PMC11976757

[19] Obermeier B Daneman R Ransohoff RM. Development, maintenance and disruption of the blood-brain barrier. Nat Med. (2013) 19:1584–96. doi: 10.1038/nm.3407 24309662 PMC4080800

[20] Terceño M Silva Y Bashir S Vera-Monge VA Cardona P Molina C . Impact of general anesthesia on posterior circulation large vessel occlusions after endovascular thrombectomy. Int J Stroke. (2021) 16:792–7. doi: 10.1177/1747493020976247 33573525

[21] Cappellari M Pracucci G Forlivesi S Saia V Nappini S Nencini P . General anesthesia versus conscious sedation and local anesthesia during thrombectomy for acute ischemic stroke. Stroke. (2020) 51:2036–44. doi: 10.1161/STROKEAHA.120.032094 32517584

[22] Mehta A Reddi P Goldman D Kellner CP De Leacy R Fifi JT . Safety and efficacy of conscious sedation versus general anesthesia for distal vessel thrombectomy. Neurosurgery. (2024) 96:104–110. doi: 10.1227/neu.0000000000003031 38856233

