# Supplementary figures and images for: Effect of general anesthesia vs. local anesthesia and collateral status on outcomes in anterior circulation occlusion
Source: Front Neurol. 2025 Nov 25;16:1665185. doi: 10.3389/fneur.2025.1665185 (PMC12685674; doi:10.3389/fneur.2025.1665185)

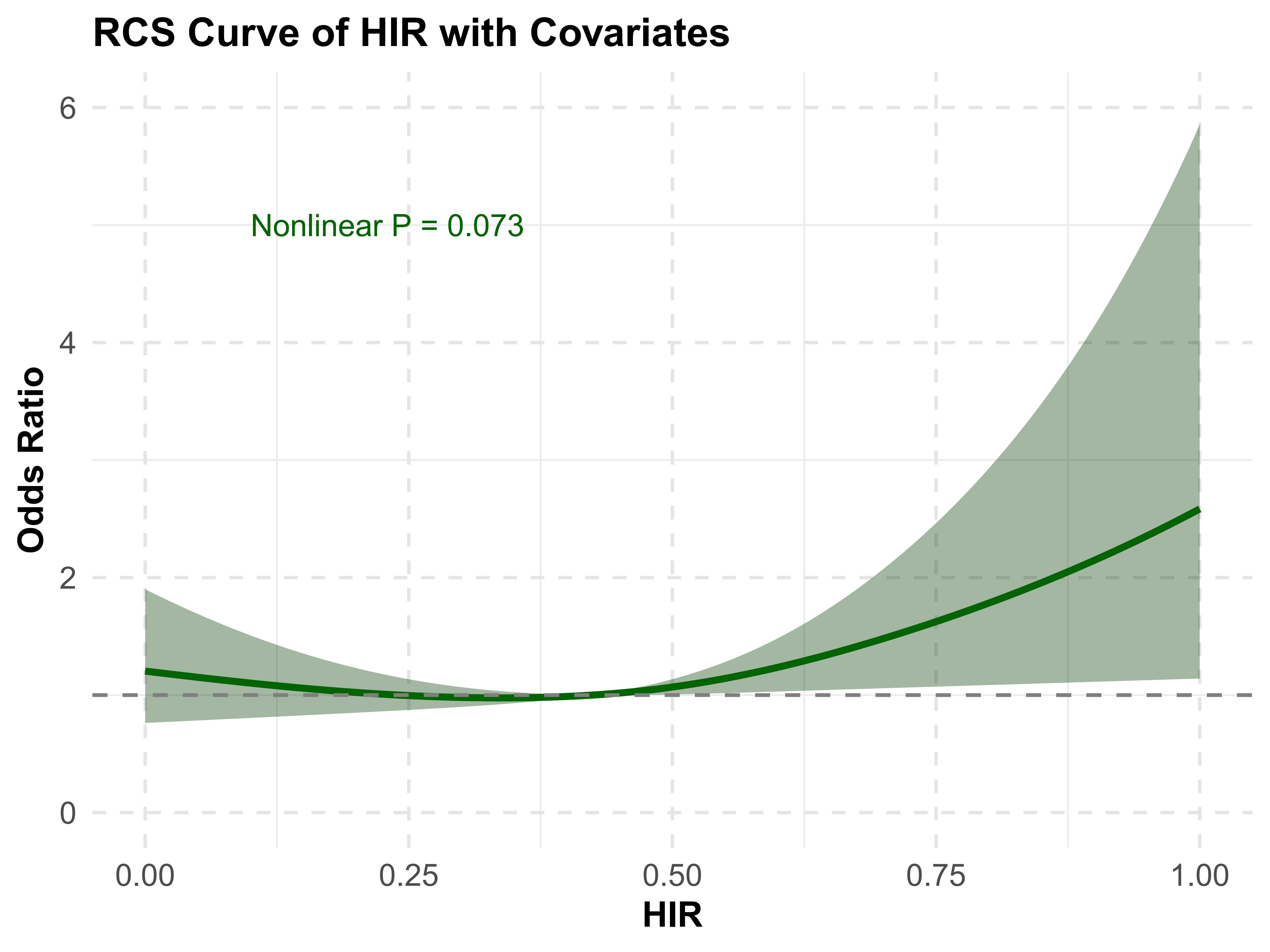

Supplement: Supplementary Figure 1 — RCS curve of HIR and primary outcome (mRS 0–2). [file Image_1.jpeg]
