# Supplementary material for: Effect of general anesthesia vs. local anesthesia and collateral status on outcomes in anterior circulation occlusion
Source: Front Neurol. 2025 Nov 25;16:1665185. doi: 10.3389/fneur.2025.1665185 (PMC12685674; doi:10.3389/fneur.2025.1665185)
Supplement: Supplementary Table 1 — Baseline characteristics before and after propensity score matching in poor collateral cohorts (HIR ≥ 0.4). [file Table_1.docx]

eTable 1. Baseline characteristics before and after propensity score matching in poor collateral cohorts (HIR≥0.4)

|  | **Before Matching** | | | |  | **After Matching** | | | |
| --- | --- | --- | --- | --- | --- | --- | --- | --- | --- |
|  | Overall  (N=375) | General anesthesia (N=218) | Local anesthesia (N=157) | SMD |  | Overall  (N=148) | General anesthesia (N=74) | Local anesthesia (N=74) | SMD |
| Demographic Data |  |  |  |  |  |  |  |  |  |
| Age, Median (IQR) | 69.00 [61.00, 77.00] | 68.00 [61.00, 75.00] | 70.00 [61.00, 77.00] | 0.057 |  | 68.50 [61.00, 75.00] | 68.00 [62.00, 74.00] | 69.00 [61.00, 77.00] | 0.041 |
| Female, (%) | 154 (41.07) | 87 (39.91) | 67 (42.68) | 0.056 |  | 58 (39.19) | 31 (41.89) | 27 (36.49) | 0.111 |
| BMI, Median (IQR) | 23.67 [21.61, 25.95] | 23.66 [21.66, 25.67] | 23.88 [21.60, 26.12] | 0.065 |  | 23.88 [21.61, 25.74] | 23.38 [21.53, 25.05] | 23.95 [21.71, 26.02] | 0.092 |
| Systolic Blood Pressure, Median (IQR) | 129.00 [119.00, 148.00] | 129.00 [118.00, 147.00] | 129.00 [120.00, 148.00] | 0.053 |  | 127.50 [120.00, 150.00] | 126.50 [119.00, 149.75] | 130.00 [120.25, 150.00] | 0.009 |
| Diastolic Blood Pressure, Median (IQR) | 78.00 [70.00, 85.00] | 79.00 [70.00, 86.00] | 77.00 [70.00, 85.00] | 0.106 |  | 79.00 [70.00, 86.00] | 79.50 [70.00, 86.00] | 78.50 [74.00, 85.00] | 0.01 |
| TOAST, (%) |  |  |  |  |  |  |  |  |  |
| Atherosclerosis | 74 (19.73) | 51 (23.39) | 23 (14.65) | 0.233 |  | 38 (25.68) | 24 (32.43) | 14 (18.92) | 0.311 |
| Cardioembolism | 183 (48.80) | 106 (48.62) | 77 (49.04) |  |  | 64 (43.24) | 31 (41.89) | 33 (44.59) |  |
| Other | 12 (3.20) | 7 (3.21) | 5 (3.18) |  |  | 4 (2.70) | 2 (2.70) | 2 (2.70) |  |
| Undetermined | 106 (28.27) | 54 (24.77) | 52 (33.12) |  |  | 42 (28.38) | 17 (22.97) | 25 (33.78) |  |
| Medical History |  |  |  |  |  |  |  |  |  |
| Wake up stroke, (%) | 81 (21.60) | 47 (21.56) | 34 (21.66) | 0.002 |  | 30 (20.27) | 16 (21.62) | 14 (18.92) | 0.067 |
| History of hypertension, (%) | 227 (60.53) | 127 (58.26) | 100 (63.69) | 0.112 |  | 88 (59.46) | 40 (54.05) | 48 (64.86) | 0.222 |
| History of diabetes mellitus, (%) | 83 (22.13) | 39 (17.89) | 44 (28.03) | 0.243 |  | 34 (22.97) | 18 (24.32) | 16 (21.62) | 0.064 |
| History of smoke, (%) | 123 (32.80) | 76 (34.86) | 47 (29.94) | 0.105 |  | 49 (33.11) | 26 (35.14) | 23 (31.08) | 0.086 |
| History of alcohol consumption, (%) | 72 (19.20) | 42 (19.27) | 30 (19.11) | 0.006 |  | 32 (21.62) | 13 (17.57) | 19 (25.68) | 0.192 |
| Previous ischemic stroke, (%) | 78 (20.80) | 41 (18.81) | 37 (23.57) | 0.117 |  | 31 (20.95) | 11 (14.86) | 20 (27.03) | 0.302 |
| History of atrial fibrillation, (%) | 162 (43.20) | 93 (42.66) | 69 (43.95) | 0.026 |  | 59 (39.86) | 27 (36.49) | 32 (43.24) | 0.138 |
| History of hyperlipemia, (%) | 68 (18.13) | 42 (19.27) | 26 (16.56) | 0.071 |  | 31 (20.95) | 17 (22.97) | 14 (18.92) | 0.1 |
| Baseline Assessments |  |  |  |  |  |  |  |  |  |
| History of coronary heart disease, (%) | 40 (10.67) | 25 (11.47) | 15 (9.55) | 0.062 |  | 15 (10.14) | 8 (10.81) | 7 (9.46) | 0.045 |
| Previous anticoagulants medication, (%) | 35 (9.33) | 19 (8.72) | 16 (10.19) | 0.05 |  | 13 (8.78) | 6 (8.11) | 7 (9.46) | 0.048 |
| Previous antiplatelet medication, (%) | 64 (17.07) | 32 (14.68) | 32 (20.38) | 0.15 |  | 25 (16.89) | 8 (10.81) | 17 (22.97) | 0.329 |
| Pre-morbidity mRS, (%) |  |  |  |  |  |  |  |  |  |
| 0 | 331 (88.27) | 196 (89.91) | 135 (85.99) | 0.089 |  | 135 (91.22) | 69 (93.24) | 66 (89.19) | 0.103 |
| 1 | 32 (8.53) | 15 (6.88) | 17 (10.83) |  |  | 9 (6.08) | 3 (4.05) | 6 (8.11) |  |
| 2 | 12 (3.20) | 7 (3.21) | 5 (3.18) |  |  | 4 (2.70) | 2 (2.70) | 2 (2.70) |  |
| Baseline NIHSS score, Median (IQR) | 17.00 [13.00, 22.00] | 18.00 [14.00, 22.00] | 16.00 [12.00, 21.00] | 0.189 |  | 16.00 [12.00, 21.00] | 16.50 [13.00, 20.75] | 16.00 [11.25, 21.00] | 0.012 |
| ASPECTS, Median (IQR) | 7.00 [5.00, 9.00] | 7.00 [5.00, 9.00] | 8.00 [6.00, 9.00] | 0.275 |  | 7.50 [6.00, 9.00] | 7.00 [6.00, 9.00] | 8.00 [5.25, 9.00] | 0.03 |
| Occlusion site, (%) |  |  |  |  |  |  |  |  |  |
| ICA | 153 (40.80) | 97 (44.50) | 56 (35.67) | 0.172 |  | 56 (37.84) | 29 (39.19) | 27 (36.49) | 0.047 |
| ACA | 6 (1.60) | 3 (1.38) | 3 (1.91) |  |  | 1 (0.68) | 1 (1.35) | 0 (0.00) |  |
| M1 | 153 (40.80) | 85 (38.99) | 68 (43.31) |  |  | 66 (44.59) | 31 (41.89) | 35 (47.30) |  |
| M2 | 61 (16.27) | 31 (14.22) | 30 (19.11) |  |  | 25 (16.89) | 13 (17.57) | 12 (16.22) |  |
| M3 | 2 (0.53) | 2 (0.92) | 0 (0.00) |  |  | 0 (0.00) | 0 (0.00) | 0 (0.00) |  |
| Ischemic core volume (mL), Median (IQR) | 36.00 [14.00, 72.00] | 43.50 [16.00, 78.75] | 29.00 [13.00, 57.00] | 0.215 |  | 29.50 [13.00, 65.50] | 29.50 [14.00, 68.00] | 29.50 [12.25, 62.25] | 0.001 |
| Mismatch volume (mL), Median (IQR) | 131.00 [91.00, 180.50] | 137.50 [92.00, 184.50] | 123.00 [85.00, 178.00] | 0.046 |  | 126.50 [91.75, 182.50] | 133.00 [86.25, 189.25] | 125.00 [93.00, 176.50] | 0.029 |
| Direct EVT | 293 (78.13) | 171 (78.44) | 122 (77.71) | 0.018 |  | 118 (79.73) | 59 (79.73) | 59 (79.73) | <0.001 |
| BGC use, (%) | 134 (35.73) | 75 (34.40) | 59 (37.58) | 0.063 |  | 53 (35.81) | 24 (32.43) | 29 (39.19) | 0.132 |
| Per procedural GPIIb IIIa receptor antagonist, (%) | 102 (27.20) | 69 (31.65) | 33 (21.02) | 0.243 |  | 46 (31.08) | 26 (35.14) | 20 (27.03) | 0.176 |
| eTICI 2c/3 on final DSA | 274 (73.07) | 150 (68.81) | 124 (78.98) | 0.233 |  | 112 (75.68) | 59 (79.73) | 53 (71.62) | 0.19 |
| Time from stroke onset to reperfusion | 395.00 [270.00, 611.50] | 409.00 [302.25, 614.00] | 349.00 [238.00, 608.00] | 0.043 |  | 376.50 [260.50, 622.25] | 412.00 [302.75, 618.75] | 328.50 [234.00, 624.75] | 0.108 |
| Outcome |  |  |  |  |  |  |  |  |  |
| mrs 0-2 at 90d, (%) | 167 (44.53) | 83 (38.07) | 84 (53.50) | 0.313 |  | 78 (52.70) | 34 (45.95) | 44 (59.46) | 0.273 |
| mrs 0-1 at 90d, (%) | 131 (34.93) | 61 (27.98) | 70 (44.59) | 0.351 |  | 61 (41.22) | 25 (33.78) | 36 (48.65) | 0.305 |
| mrs 0-3 at 90d, (%) | 206 (54.93) | 111 (50.92) | 95 (60.51) | 0.194 |  | 91 (61.49) | 42 (56.76) | 49 (66.22) | 0.195 |
| Symptomatic intracranial hemorrhage, (%) | 22 (5.87) | 15 (6.88) | 7 (4.46) | 0.105 |  | 10 (6.76) | 5 (6.76) | 5 (6.76) | <0.001 |
| NIHSS score at 7 days | 8.00 [3.00, 19.00] | 10.00 [3.25, 20.00] | 5.00 [2.00, 17.00] | 0.268 |  | 6.00 [2.00, 18.25] | 7.00 [3.00, 18.75] | 4.50 [2.00, 17.75] | 0.067 |
| stroke associated pneumonia, (%) | 126 (33.60) | 85 (38.99) | 41 (26.11) | 0.277 |  | 39 (26.35) | 21 (28.38) | 18 (24.32) | 0.092 |
| Early neurological deterioration, (%) | 55 (14.67) | 41 (18.81) | 14 (8.92) | 0.289 |  | 17 (11.49) | 10 (13.51) | 7 (9.46) | 0.127 |
| Mortality at 90d, (%) | 67 (17.87) | 47 (21.56) | 20 (12.74) | 0.236 |  | 21 (14.19) | 11 (14.86) | 10 (13.51) | 0.039 |
| Values are presented as mean (standard deviation) or median [Q1, Q3] for continuous variables and number (percentage) for categorical variables. Variables in bold have p-value < 0.05. | | | | | | | | | |

eTable 2. Secondary outcome (mRS 0-1) with different models for cohort.

|  | **Collateral status** | **p-value** | **OR (95 CI) 1** |
| --- | --- | --- | --- |
| Propensity score matching | Good | <0.05 | 2.78 (1.30, 5.95) |
|  | Poor | 0.118 | 1.73 (0.92, 3.27) |
| Multivariate logistic model adjusted with all covariates | Good | <0.01 | 2.13 (1.31, 3.51) |
|  | Poor | 0.052 | 1.66 (1.00, 2.76) |
| Propensity score IPTW | Good | <0.001 | 2.08 (1.51, 2.87) |
|  | Poor | <0.01 | 1.65 (1.17, 2.33) |
| Doubly robust estimation with all covariates | Good | <0.01 | 2.08 (1.26, 3.41) |
|  | Poor | 0.056 | 1.65 (0.99, 2.75) |
| Doubly robust estimation with unbalanced covariates | Good | <0.01 | 2.03 (1.24, 3.32) |
|  | Poor | 0.054 | 1.65 (0.99, 2.75) |
|  | Statistical analyses of different models with p-value < 0.05 were displayed in bold. | | |
|  | ^1^OR = Odds Ratio, CI = Confidence Interval | | |

eTable 3. Secondary outcome (mRS 0-3) with different models for cohort.

|  | **Collateral status** | **p-value** | **OR (95 CI) 1** |
| --- | --- | --- | --- |
| Propensity score matching | Good | <0.01 | 4 (1.50, 10.66) |
|  | Poor | 0.324 | 1.47 (0.76, 2.83) |
| Multivariate logistic model adjusted with all covariates | Good | <0.01 | 2.53 (1.45, 4.55) |
|  | Poor | 0.345 | 1.27 (0.78, 2.07) |
| Propensity score IPTW | Good | <0.001 | 2.79 (1.91, 4.11) |
|  | Poor | 0.136 | 1.28 (0.93, 1.78) |
| Doubly robust estimation with all covariates | Good | <0.001 | 2.79 (1.59, 4.89) |
|  | Poor | 0.325 | 1.28 (0.78, 2.11) |
| Doubly robust estimation with unbalanced covariates | Good | <0.001 | 2.79 (1.59, 4.89) |
|  | Poor | 0.325 | 1.28 (0.78, 2.11) |
|  | Statistical analyses of different models with p-value < 0.05 were displayed in bold. | | |
|  | ^1^OR = Odds Ratio, CI = Confidence Interval | | |

eTable 4. Analysis results of the secondary outcomes of the cohort.

|  | **Collateral status** | **p-value** | **OR (95 CI) 1** |
| --- | --- | --- | --- |
| Stroke associated pneumonia | Good | 0.188 | 1.62 (0.79, 3.32) |
|  | Poor | <0.05 | 2.37 (1.21, 4.66) |
| Early neurological deterioration | Good | <0.05 | 4.40 (1.43, 13.50) |
|  | Poor | <0.05 | 2.76 (1.10, 6.93) |
| Mortality at 90d | Good | 0.222 | 1.97 (0.66, 5.85) |
|  | Poor | <0.05 | 2.94 (1.20, 7.21) |
| Hemorrhagic transformation | Good | 0.279 | 1.38 (0.77, 2.46) |
|  | Poor | 0.101 | 1.68 (0.90, 3.12) |
| Statistical analyses of multivariate logistic model with p-value < 0.05 were displayed in bold.  ^1^OR = Odds Ratio, CI = Confidence Interval | | | |
